# Stalking syndrome in clinical forensic psychiatry

**DOI:** 10.1192/j.eurpsy.2025.1524

**Published:** 2025-08-26

**Authors:** J. Sredanovic, L. Cuk- Jovanovic, E. Novakovic, J. Tomasevic, T. Novakovic

**Affiliations:** 1 Clinic for mental disorders dr Laza Lazarevic, Belgrade; 2 Faculty of Medicine, University in Priština, Kosovska Mitrovica, Serbia

## Abstract

**Introduction:**

This study is probably the first study on stalking conducted in the Republic of Serbia.

**Objectives:**

The aim of this study was to examine the stalking experiences of a sample of persons who, according to the Court’s judgment, were in need of treatment.

**Methods:**

This retrospective study was conducted from January 2020 until January 2024 and included 46 persons on the measure of treatment treated at the Clinic for Mental Disorders “Dr Laza Lazarević” in Belgrade. All obtained data were from their medical records and based on the judgment of the Court. The data were processed using SPSS version 21 to produce mainly descriptive and inferential statistics. Difference were considered statistically significant if p< 0.05.

**Results:**

The participantes were mean age 49.5 ±12.9 years, and most of them 41 (89.1%) were men. The stalker was in most cases a male, he was unemployed (65.2%), unmarried (91,3%), lived with his parents (67.4%) in the city (91.3%). Stalkers were most often diagnosed with F22 (23.9%) and F23 (23.9%). The stalker with the diagnosis F22 most often pursued the desired partner (33.3%) and the stalker with the diagnosis F23 a person from the social environment (33.3%). After the treatment measure is completed, the stalker often repeats the same act (12.83%).

**Conclusions:**

Stalking remains a major problem and insufficiently tested that must be taken seriously. It is best to look at stalkers as a heterogeneous group whose behavior can be motivated by various psychiatric illnesses, predominantly psychoses.

**Disclosure of Interest:**

None Declared

